# EHMT2 (G9a) activation in mantle cell lymphoma and its associated DNA methylation and gene expression

**DOI:** 10.20892/j.issn.2095-3941.2020.0371

**Published:** 2021-10-12

**Authors:** Jun Wang, Hui Xu, Shuang Ge, Chaoshuai Xue, Hailing Li, Xiaotong Jing, Ke Liang, Xiaoying Zhang, Cuijuan Zhang

**Affiliations:** 1 Institute of Pathology and Pathophysiology, Shandong University School of Medicine, Jinan 250012, China; 2 Department of Pathology, Qianfoshan Hospital, Jinan 250300, China; 3 Department of Pathology, Qilu Hospital of Shandong University, Jinan 250012, China

**Keywords:** DNA methylation, mantle cell lymphoma, epigenetic biomarker, EHMT2, gene expression

## Abstract

**Objective:**

The function of euchromatic histone-lysine N-methyltransferase 2 (EHMT2) has been studied in several cancers; however, little is known about its role in mantle cell lymphoma (MCL). Thus, this study aimed to characterize the significance and function of EHMT2 in MCL.

**Methods:**

EHMT2 expression in MCL and reactive hyperplasia (RH) were investigated by immunohistochemistry. Genome-wide analysis of DNA methylation was performed on EHMT2 + MCL samples. The function of EHMT2 was determined by CCK8, flow cytometry, and western blot assays. Gene expression profile analysis was performed before and after *EHMT2* knockdown to search for EHMT2-regulated genes. Co-immunoprecipitation (Co-IP) experiments were conducted to identify the proteins interacting with EHMT2.

**Results:**

EHMT2 was expressed in 68.57% (24/35) of MCLs but not in any RHs. Genome-wide analysis of DNA methylation on EHMT2 + MCLs revealed that multiple members of the *HOX*, *FOX*, *PAX*, *SOX*, and *CDX* families were hypermethylated or hypomethylated in EHMT2 + MCLs. BIX01294, a EHMT2 inhibitor, inhibited MCL cell growth and stalled cells in the G1 phase. Additionally, BIX01294 downregulated the expressions of cell cycle proteins, cyclin D1, CDK4, and P21, but upregulated the expressions of apoptosis-related proteins, Bax and caspase-3. Co-IP experiments revealed that EHMT2 interacted with UHRF1, HDAC1, and HDAC2 but not with HDCA3. After *EHMT2* knockdown, multiple genes were regulated, including *CD5* and *CCND1*, mostly enriched in the Tec kinase signaling pathway. In addition, several genes (e.g., *MARCH1*, *CCDC50*, *HIP1*, and *WNT3*) were aberrantly methylated in EHMT2 + MCLs.

**Conclusions:**

For the first time, we determined the significance of EHMT2 in MCL and identified potential EHMT2-regulated genes.

## Introduction

Mantle cell lymphoma (MCL) is a clinically aggressive, incurable B-cell malignancy, accounting for 5% of non-Hodgkin lymphomas (NHLs), with a median survival of 3–4 years. It is characterized by the presence of a t (11;14) (q13;q32) chromosomal translocation with the juxtaposition of the *CCND1* gene to the IGHV locus, resulting in the overexpression of cyclin D1. However, murine models overexpressing cyclin D1 in the absence of other oncogenes, such as myc, do not develop lymphoma, implying that additional mechanisms are involved in MCL^1,2^.

Epigenetic changes, such as DNA methylation and histone modifications, have been shown to contribute to the pathogenesis of lymphomas. DNA methylation and histone modifications often functionally cooperate to repress transcription. Euchromatic histone-lysine N-methyltransferase 2 (EHMT2, also known as G9a), is a histone methyltransferase that mediates the methylation of histones H3K9-me1, H3K9-me2, and H3K27, and plays a crucial role in gene silencing^3–6^. EHMT2 was first identified as a gene located in the major histocompatibility complex (MHC) locus in mice and the human leukocyte antigen (HLA) locus in humans^7,8^. It was reported that EHMT2 mediated DNA methylation, and this process may be independent of its H3K9 methyltransferase activity^9,10^. EHMT2 overexpression is present in various cancers, including esophageal squamous cell carcinoma^11^, hepatocellular carcinoma^12^, lung cancer^13^, breast cancer^14^, and ovarian cancer^15^. Elevated EHMT2 levels are commonly correlated with higher methylation levels, leading to the suppression of important tumor suppressor genes^16^. EHMT2 depletion has been reported to inhibit cell proliferation in several cancer cell lines^17,18^. In lymphohematopoietic tumors, loss of EHMT2 significantly delayed disease progression and reduced leukemia stem cell frequency in an acute myeloid leukemia mouse model^19^. However, very little is known about the role of EHMT2 in MCL.

In this study, we showed that EHMT2 was overexpressed in MCL patients compared with reactive hyperplasia (RH) patients. Given that EHMT2-associated specific methylation of genes might be a critical step in MCL lymphomagenesis, we performed genome-wide analysis of DNA methylation on EHMT2 + MCL patients using RH cases as the controls to obtain a more comprehensive understanding of aberrant DNA methylation in MCL. We also treated MCL cells with an EHMT2 inhibitor, BIX01294, to verify the functions of EHMT2 in MCL. Subsequently, we used gene expression microarrays to investigate the EHMT2-regulated genes after knockdown of *EHMT2*. By comparing the gene expression profiles (GEPs) with the DNA methylation profiles in EHMT2 + MCL, we identified several aberrantly methylated genes whose expressions were regulated by EHMT2. The E3 ubiquitin ligase, ubiquitin-like, containing PHD and RING finger domains, 1 (UHRF1), is a master regulator of epigenetic modifications due to its ability to recognize modifications of both DNA and histones. By recognizing hemi-methylated DNA, UHRF1 maintains genomic DNA methylation and regulates gene expression with histone deacetylase by binding to methylated histones. EHMT2 is recruited to the *UHRF1* promoter and functions as a corepressor of target genes^20,21^. In the current study, we also investigated the interactions of EHMT2 with UHRF1, HDAC1, HDAC2, and HDAC3 in MCL cells. For the first time, our data identified the significance of EHMT2 in MCL, and proposed possible mechanisms of EHMT2-associated DNA methylation and gene expression.

## Materials and methods

### Patient samples and clinical data

In this study, 35 MCL samples were collected from Qilu Hospital of Shandong University between 2006 and 2017. The patient ages ranged from 25 to 81 years (median: 59.8 years), and the male-to-female ratio was 27:8. All cases were reviewed independently by 2 pathologists to confirm the diagnoses. Diagnoses were classified according to the World Health Organization Classification 2017. Written informed consent was obtained from all patients, and necessary approvals were obtained from the Shandong University Ethics Committee.

### Immunohistochemistry

Immunohistochemistry was performed on 5 μm formalin-fixed, paraffin-embedded tissue sections according to standard methods. Briefly, the slides were deparaffinized in xylene and rehydrated in an ethanol gradient. Following antigen retrieval, the slides were incubated sequentially with 3% H_2_O_2_, goat serum, and anti- EHMT2 primary antibody (1:100, Abcam, Cambridge, MA, USA) overnight at 4 °C. Thereafter, the slides were incubated with horseradish peroxidase (HRP)-conjugated secondary antibody for 30 min at 37 °C. Then, 3,3-diaminobenzidine (Vectastain Laboratory, Burlingame, CA, USA) was used as a chromogen, and Meyer’s hematoxylin was used for counterstaining. EHMT2 was considered positive when the tumor cells exhibited distinct nuclear staining.

### DNA methylation array

The DNA methylation profiling was performed on 4 EHMT2 + MCL and 4 RH bases using the Illumina Infinium Human Methylation 850K (HM850K) Bead-Chip array (Illumina, San Diego, CA, USA), which targeted >85,000 CpG sites spanning over 42,000 genes. A Genomic DNA Purification Kit (Qiagen, Hilden, Germany) was used to extract genomic DNA according to the manufacturer’s instructions. All samples were processed on the same chip to avoid chip-to-chip variations.

### Cell culture and drug treatment

The Jeko-1 and Mino MCL cell lines were obtained from the American Type Culture Collection (ATCC; Rockville, MD, USA). The cell lines had been authenticated using short tandem repeat (or single nucleotide polymorphism) profiling in 2019. The cells were maintained in RPMI-1640 with 15% fetal bovine serum at 37 °C and 5% CO_2_. Jeko-1 and Mino cells were treated with different concentrations of BIX01294 (Sigma-Aldrich, St. Louis, MO, USA) for 48 h to observe changes in cell function. All experiments were performed using mycoplasma-free cells.

### Cell proliferation assay

Cell Counting Kit-8 (CCK-8; Dojindo, Tokyo, Japan) was used to examine cell proliferation according to the manufacturer’s instructions. A cell suspension was inoculated into 96-well plates (2 × 10^4^ cells/well) and incubated for 24 h. For the cell viability assays, the cells were seeded into 96-well plates and exposed to various concentrations of BIX01294 (Jeko-1: 1, 2, 4, or 8 μmol; Mino: 0, 1, 2, 4, or 6 μmol) for 0, 24, 48, 72, or 96 h. At each time point, 10 μL of CCK-8 was added to each well and the plate was incubated for 3 h at 37 °C and 5% CO_2_. The absorbance was measured at 450 nm using a microplate reader. The assay was performed using 6 replicates (*n* = 6) for each group and was repeated at least 3 times.

### Flow cytometry

Mino and Jeko-1 cells treated with different concentrations of BIX01294 were cultured in 6-well plates for 48 h. For cell cycle analysis, the cells were harvested and fixed in 70% ethanol overnight at −20 °C before staining with propidium iodide (PI; BestBio, Shanghai, China). To evaluate cell apoptosis, the cells were washed with Annexin V binding buffer and stained with Annexin V-fluorescein isothiocyanate (FITC) and PI, using the FITC Annexin V Apoptosis Detection kit (BestBio). The harvested cells were analyzed by flow cytometry (CytoFLEX; Beckman Coulter, Shanghai, China) according to the manufacturer’s recommendations.

### Western blotting

All sample protein extracts were separated by 10% SDS-PAGE and transferred to polyvinylidene difluoride membranes. After blocking for 2 h in Tris-buffered saline with 0.1% Tween^®^ 20 detergent (TBST) containing 5% nonfat milk, the membranes were incubated with primary antibodies diluted in TBST containing 5% nonfat milk at 4 °C overnight. The following primary antibodies were used: mouse anti-glyceraldehyde 3-phosphate dehydrogenase (PMK Biotechnology, Shijiazhuang City, China); mouse anti-cyclinD1 (Cell Signaling Technology, Danvers, MA, USA); mouse anti-CDK4 (Cell Signaling Technology); mouse anti-P21 (Cell Signaling Technology); mouse anti-Bax (Cell Signaling Technology); and mouse anti-caspase-3 (Abcam, Shanghai, China). The membranes were washed 3 times with TBST and incubated with HRP-conjugated secondary antibodies for 2 h at room temperature. They were then washed 3 times with TBST and the signals were visualized using ECL plus reagents (Amersham Biosciences, Buckingham, UK).

### *EHMT2* short hairpin RNA (shRNA) knockdown and cell transfection

The* EHMT2* shRNA plasmids were purchased from GenePharma (Shanghai, China). The sequences used for *EHMT2* knockdown were as follows: *EHMT2*-homo-278, GAG AGA GTT CAT GGC TCT TTG; *EHMT2*-homo-2188, GCA GGC TGG AGC CAA CAT AAA; *EHMT2*-homo-2766, GGT TTG CGC TTC AAC TCA ACC; and *EHMT2*-homo-2910, GCC CTG AGG ATT ACA AGT ACA. Mino cells were transfected with shRNA, using Lipofectamine^®^ 2000 (Invitrogen, Carlsbad, CA, USA). The transfection procedures were performed according to the manufacturer’s protocol.

### Co-immunoprecipitation (Co-IP)

Immunoprecipitation of EHMT2 was performed using the Pierce Crosslink Immunoprecipitation Kit (Pierce/Thermo Fisher Scientific, Waltham, MA, USA) according to the manufacturer’s instructions. Briefly, cells were lysed in ice-cold immunoprecipitation lysis/wash buffers. Next, 10 μL was removed for use as the input, and the remainder was divided into 2 equal parts, each incubated with antibody (anti-IgG as the nonspecific negative control) overnight at 4 °C. The immunoprecipitate was washed 3 times with cold lysis buffer, and then eluted with 10 μL of elution buffer. After centrifugation, the supernatant was analyzed by western blotting. The following commercial antibodies were used against: EHMT2 (Abcam); UHRF1 (Active Motif, Carlsbad, CA, USA); and HDAC1, HDAC2, and HDAC3 (Abcam).

### *EHMT2* short hairpin RNA (shRNA) knockdown

The *EHMT2* shRNA plasmids were purchased from GenePharma. Jeko-1 and Mino cells were transfected with shRNA using Lipofectamine^®^ 2000 (Invitrogen). The transfection procedures were performed according to the manufacturer’s protocol. The sequences used for *EHMT2* shRNA knockdown were as follows: *EHMT2*-homo-278, GAGAGAGTTCATGGC TCTTTG; *EHMT2*-homo-2188, GCAGGCTGGAGCCAACATAAA; *EHMT2*-homo-2766, GGTTTGCGCTTCAACTCAACC; and *EHMT2*-homo-2910, GCCCTGAGGATTACAAGTACA.

### Gene expression profiles

The *EHMT2*-knockdown and control cells were collected, and total RNA was extracted using TRIzol reagent and purified using a Qiagen RNeasy Mini Kit. The total RNA samples were analyzed using the Agilent 2100 system (Agilent, San Jose, CA, USA), and aRNA (amplified RNA) was prepared using the GeneChip 3′IVT Express Kit (Thermo Fisher Scientific). Next, aRNA was purified, fragmented, and hybridized with the microarray probes. After hybridization, the arrays were washed, dyed, and scanned. The bioconductor “limma” package (http://bioconductor.org) was used to analyze the microarray data. Background-corrected data were log_2_ transformed and quantile-normalized. Moderated *t*-statistics were used to test whether genes were differentially expressed between *EHMT2* knockdown and control cells. The Benjamini-Hochberg method was used to estimate the false discovery rate (FDR). FDR < 0.05 was considered statistically significant.

### Statistical analysis

Statistical analysis was performed using SPSS statistical software for Windows, version 18.0 (SPSS, Chicago, IL, USA). The -values were used to calculate the percent difference in methylation between MCL and RH, which ranged from 0 (unmethylated) to 1 (100% methylated). The data are expressed as the mean ± SD of triplicates for each experiment. Differences between groups were evaluated using analysis of variance. *P* < 0.05 was considered to be statistically significant.

## Results

### EHMT2 expression in MCLs compared with that in RHs

**Figure 1:**
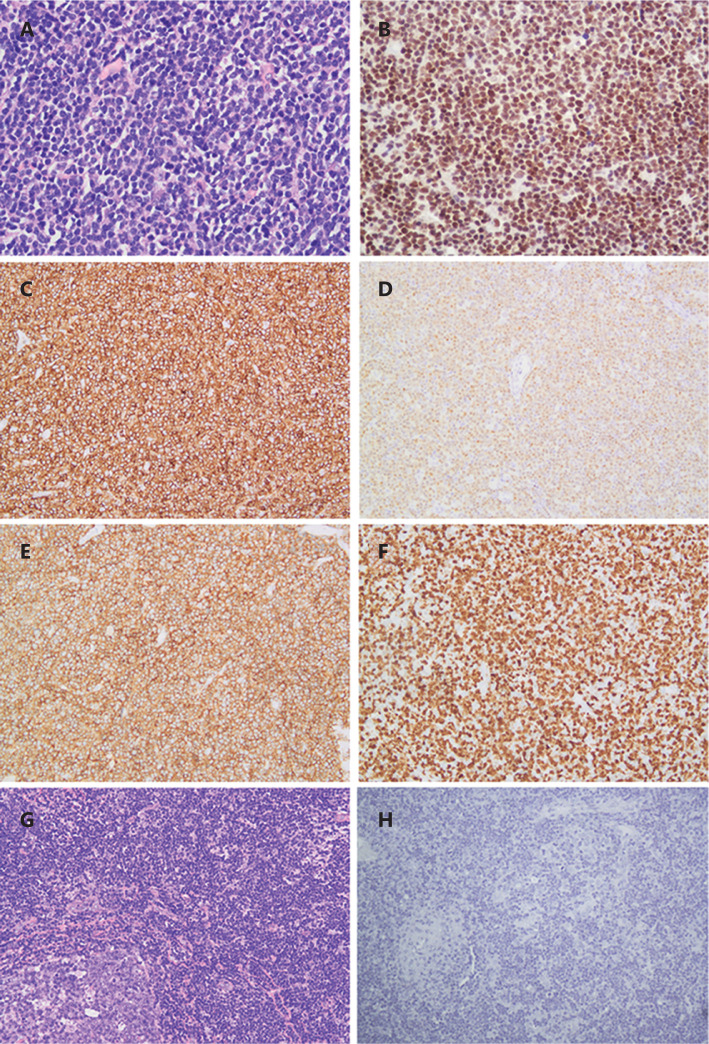
EHMT2 expression in mantle cell lymphoma (MCL) and reactive hyperplasia (RH) cases. A, Hematoxylin and eosin (H&E) staining of MCL, 400×; B, EHMT2 expression in MCL, 400×; C, CD20 expression in MCL, 200×; D, cyclinD1 expression in MCL, 200×; E, CD5 expression in MCL, 200×; F, Ki67 expression in MCL, 200×; G, H&E staining of RH, 200×; H, EHMT2 expression in RH, 200×.

In the present study, we investigated expressions of EHMT2 protein in 35 cases of MCL and 20 cases of RH by immunohistochemistry. EHMT2 was diffusely positive in 22 (68.57%, 24/35) cases of MCL but was negative in all cases of RH (**Figure 1**). To evaluate the biological significance of EHMT2 in MCL, we assessed associations between EHMT2 expression and the clinicopathological features of MCL (**Supplementary Table S1**). EHMT2 was overexpressed both in tumors (17/22, 77.27%) with a low Ki67 index and those with a high Ki67 index (7/13, 53.85%). No significant association was detected between EHMT2 expression and sex, age, levels of LDH, or Ann Arbor Stage in patients.

### DNA methylation profiles in EHMT2+ MCLs compared with that in RHs

**Figure 2:**
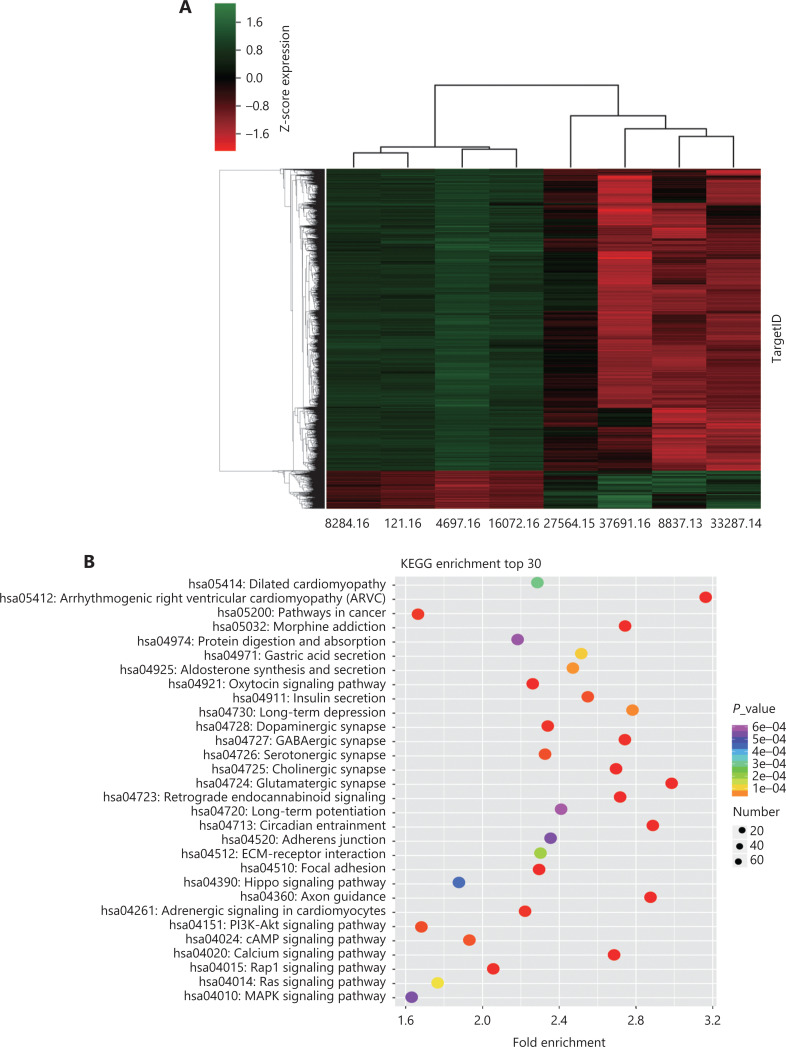
(A) Heat map showing an overview of the hierarchical clustering of differentially methylated genes in EHMT2 + mantle cell lymphomas (MCLs) and controls. Hypermethylation is denoted by red, and hypomethylation is denoted by green. (B) Kyoto Encyclopedia of Genes and Genomes (KEGG) analysis of the differentially methylated genes in EHMT2 + MCL cases (β > 0.14; *P* < 0.05). Red denotes smaller *P*-values, and green denotes larger values. The x-axis represents the enrichment factor, and the y-axis represents the KEGG category. The top 30 KEGG terms are shown.

To identify which genes exhibited aberrant methylation in activated EHMT2 cases, we performed genome-wide DNA methylation detection of EHMT2 + MCLs compared with that of RHs. In all 47,952 CpG sites (covering 13,688 genes), 10,133 CpG sites (covering 7,235 genes) (21.2%) significantly differed between EHMT2 + MCL and RH cases using delta_beta ≥0.3. The heat map of differential methylation is shown in **Figure 2A**. Among all significant CpG sites, 1,338 (13.20%) were significantly hypermethylated (covering 674 genes), and 8,795 (86.80%) were significantly hypomethylated (covering 6,561 genes) in EHMT2 + MCLs cases (**Supplementary Table S2**). The genomic regions of the significantly hyper- or hypomethylated CpG sites were differentially distributed (**Supplementary Table S3**) and the biological characteristics of significant CpG sites identified in MCLs are shown in **Supplementary Table S4**. A total of 83.11% (1,112) of significantly hypermethylated CpGs were in CpG island regions, with 12.03% (161) in CpG shores, 1.12% (15) in shelf regions, and 3.74% (50) in open sea regions. Conversely, only a small proportion (1.11%) of the significantly hypomethylated CpGs (98) were found in islands, while most were found in open sea (84.02%) regions. The genomic distribution of significant CpG sites revealed that 486 (36.32%) significantly hypermethylated CpGs were located within promoter regions. However, most of the hypomethylated CpG sites were located within intergenic (42.55%) or body (40.94%) regions; only 8.62% (758) were located within promoter regions.

Gene ontology (GO) and Kyoto Encyclopedia of Genes and Genomes (KEGG) analyses were also performed to identify the biological pathways of differentially methylated genes in EHMT2 + MCL cases. The methylated genes were significantly enriched in anabolism, organ development, and transmembrane transport (**Supplementary Figure S1**). KEGG analysis revealed that several pathways, including PI3K-Akt, MAPK, Rap1, cAMP, and calcium signaling pathways were involved in gene methylation-related carcinogenesis (**Figure 2B**). Notably, multiple members of the HOX genes (*n* = 15) (*HOXA7*, *HOXA9*, *HOXA10*, *HOXA11*, *HOXA13*, *HOXB8*, *HOXC11*, *HOXC12*, *HOXC13*, *HOXD1*, *HOXD8*, *HOXD9*, *HOXD11*, *HOXD12*, and *HOXD13*); FOX genes (*n* = 13) (*FOXA1*, *FOXA2*, *FOXB1*, *FOXC1*, *FOXC2*, *FOXD1*, *FOXD3*, *FOXE1*, *FOXE3*, *FOXF2*, *FOXG1*, *FOXL2*, and *FOXQ1*); SOX genes (*SOX2*, *SOX9*, and SOX17); and PAX genes (*PAX1*, *PAX3*, *PAX6*, and *PAX7*) were frequently methylated in EHMT2 + MCLs. Notably, several other SOX genes (*SOX2*, *SOX5*, *SOX6*, and *SOX11*) and PAX (*PAX3* and *PAX5*) genes were hypomethylated. **Supplementary Tables S5 and S6** show the top 30 significantly hypomethylated and hypermethylated genes in EHMT2 + MCL cases, when compared with those in RH cases.

### Effects of the EHMT2 inhibitor, BIX01294, on MCL cells

**Figure 3:**
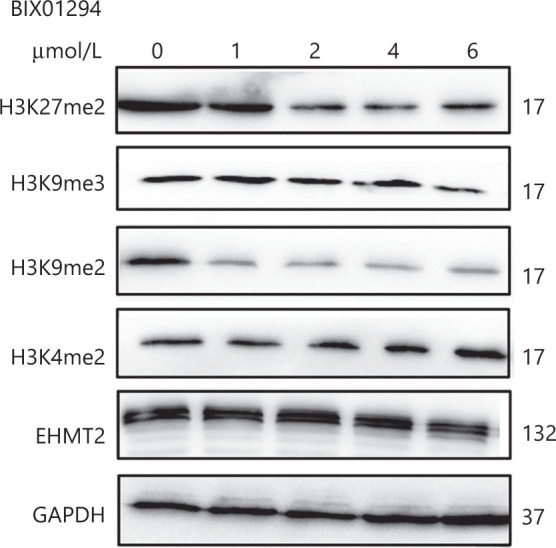
Changes in the H3K4, H3K9, and H3K27 methylations and EHMT2 protein levels after treatment with the EHMT2, inhibitor BIX01294. Note that H3K9me2 and H3K27me2 levels decreased with increase in drug concentration; conversely, H3K4me2 levels increased. H3K9me3 and EHMT2 showed no changes after treatment with BIX01294.

To evaluate the effects of EHMT2 on MCL cells, we treated MCL cells with an EHMT2 inhibitor, BIX01294. With increasing BIX01294 concentrations, the H3K9me2 and H3K27me2 levels decreased, while the H3K4me2 levels increased; the H3K9me3 levels were unaffected (**Figure 3**). The EHMT2 protein level showed no change after treatment with BIX01294 in MCL cells, indicating that BIX01294 regulated histone methylation by inhibiting the activation of EHMT2, but not its expression.

**Figure 4:**
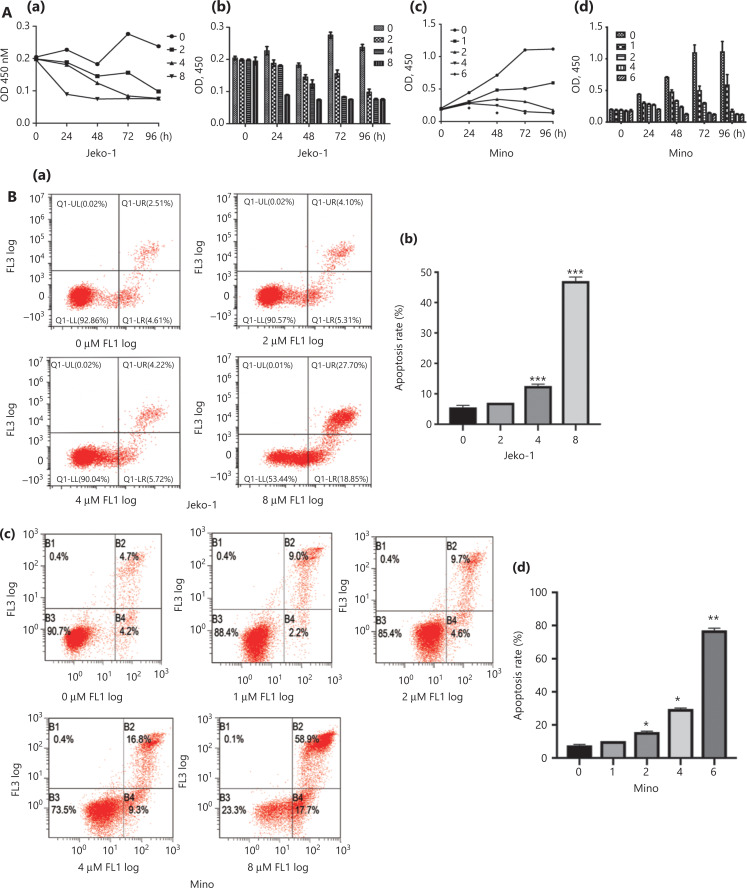

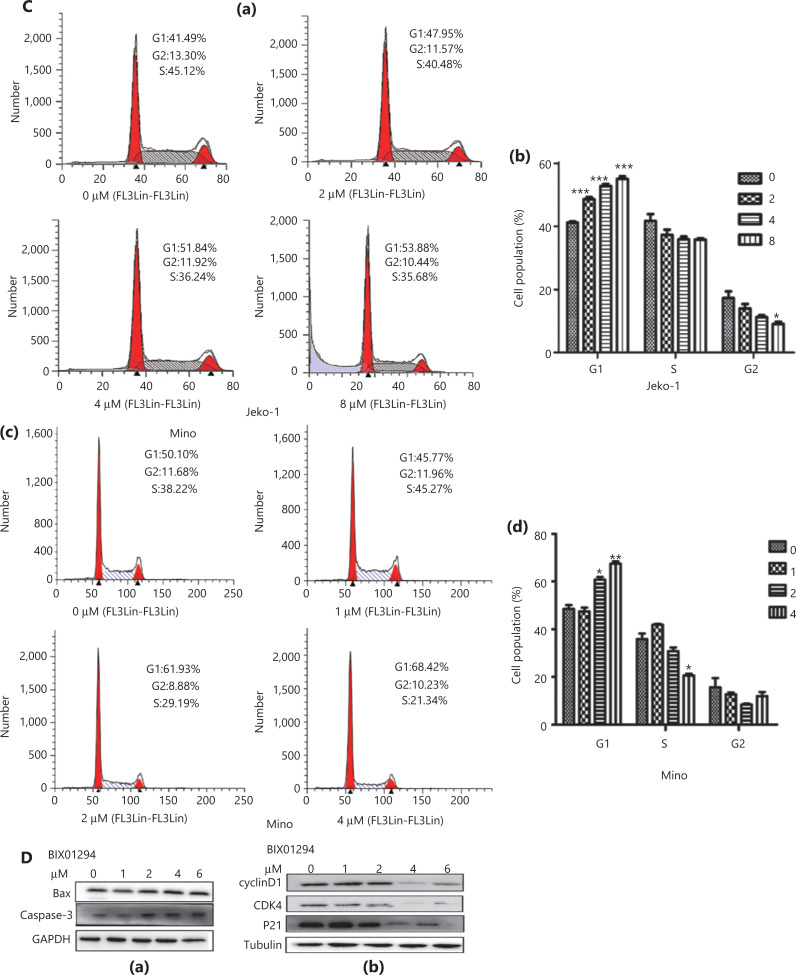
The effects of the EHMT2 inhibitor, BIX01294, on mantle cell lymphoma cell proliferation (A), cell apoptosis (B), the cell cycle (C), and the expression of related proteins (D). A: (a, c) Survival curves of Jeko-1 and Mino cells after treatment with different concentrations of BIX01294; (b, d) Column diagram of the cell proliferation of Jeko-1 and Mino cells after treatment with different concentrations of BIX01294. B: (a, c) Apoptosis of Jeko-1 and Mino cells after treatment with different concentrations of BIX01294; (b, d) Apoptosis rate of Jeko-1 and Mino cells after treatment with different concentrations of BIX01294. C: (a, c) Number of cells (Jeko-1 or Mino) in different stages of the cell cycle after treatment with different concentrations of BIX01294; (b, d) Cell populations in different cell cycle stages after treatment with different concentrations of BIX01294. D: (a) Expression of apoptosis-related proteins, Bax and caspase-3, after treatment with different concentrations of BIX01294; (b) Expression of cell cycle-related proteins, cyclinD1, CDK4, and CD21, after treatment with different concentrations of BIX01294. *P < 0.05, **P < 0.01 and ***P < 0.001. The cells treated with different concentrations of BIX01294 were compared with those treated without BIX01294.

After treatment with BIX01294, MCL proliferation was significantly inhibited, depending on the concentration and duration of treatments (**Figure 4A**). Accordingly, flow cytometry showed a concentration- and time-dependent increase in apoptotic cells (**Figure 4B**). Furthermore, BIX01294-treated cells exhibited significantly more cells arrested in G0/G1 than untreated cells, and fewer cells in G2/M and S phases (**Figure 4C**). Additionally, the cell cycle proteins, cyclin D1, CDK4, and p21 were downregulated, while apoptosis-related proteins, Bax and capase-3, were upregulated after treatment with different concentrations of BIX01294 (**Figure 4D**).

### Interaction of EHMT2 with UHRF1 and histone deacetylases (HDACs)

**Figure 5:**
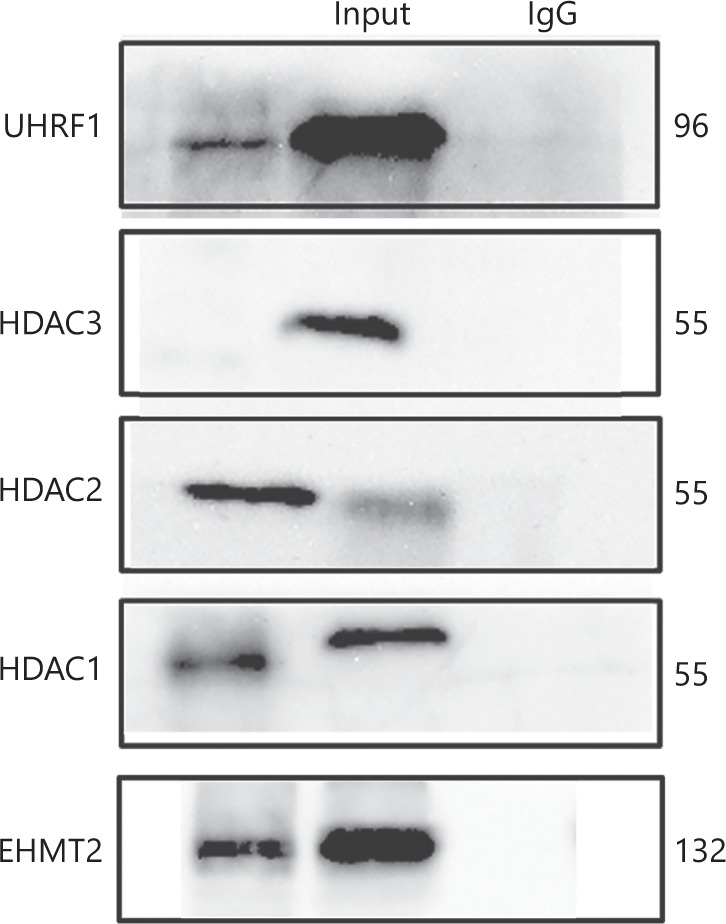
Interaction of EHMT2 with UHRF1 and HDACs. The schematic indicates that EHMT2 interacts with UHRF1, HDAC1, and HDAC2, but not with HDAC3.

Because EHMT2 lacks a DNA-binding domain, it depends on additional cofactors for its localization at specific genetic loci. Recently, EHMT2 was reported to bind UHRF1, and both were co-localized in the nucleus in a cell-cycle-dependent manner. To investigate whether EHMT2 interacted with UHRF1 in MCL, we performed Co-IP experiments to detect their possible interaction. Given that histone methyltransferases often cooperate with HDACs during epigenetic regulation, we also tested the interactions between EHMT2 and HDAC1/2/3. EHMT2 was bound to UHRF1, and also to HDAC1 and HDAC2. However, no binding of EHMT2 to HDAC3 was detected, suggesting that EHMT2 contributed to MCL by interacting with UHRF1, HDAC1, and HDAC2, while HDAC3 did not participate in this process (**Figure 5**).

### GEP analyses identified the target genes regulated by *EHMT2* in MCL

**Figure 6:**
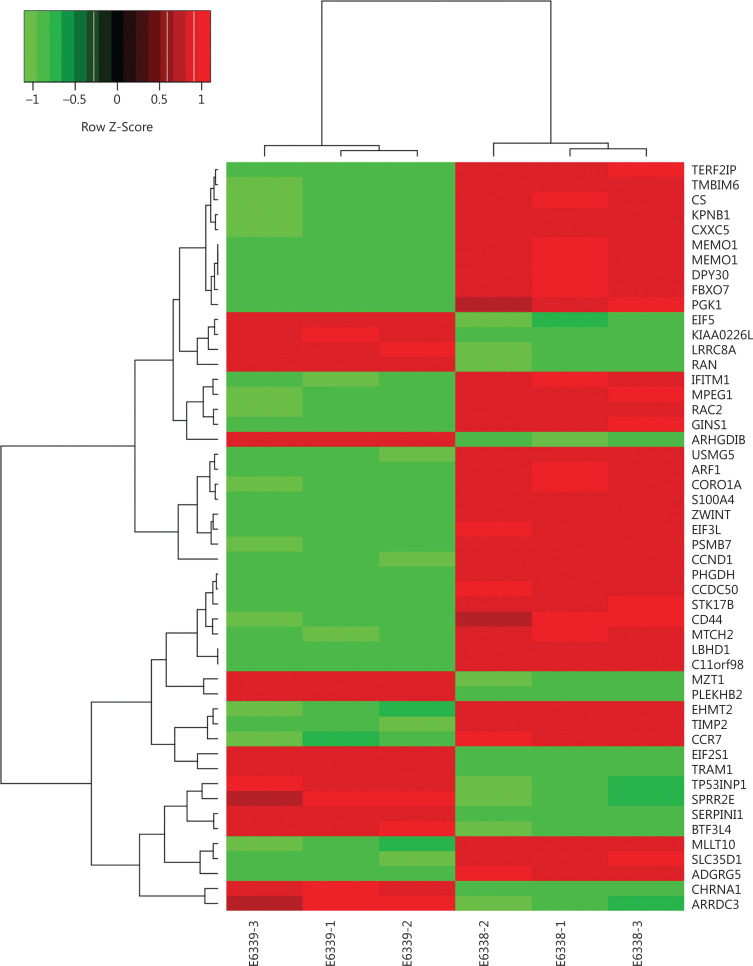
Part of a heat map of target genes regulated by EHMT2 in mantle cell lymphomas (MCLs). E6338-1, E6338-2, and E6338-3 indicate MCL cells without knockdown of *EHMT2*; E6339-1, E6339-2, and E6339-3 indicate MCL cells after *EHMT2* knockdown; Red denotes upregulation. Green denotes downregulation. Black denotes no change. The right column shows the top 30 genes that were significantly downregulated and upregulated after *EHMT2* knockdown.

To search for target genes regulated by EHMT2 in MCL, we performed GEP analysis on MCL cells before and after *EHMT2* knockdown. Genes were considered to be differentially expressed if their expression showed at least a 1.5-fold change and an FDR < 0.05. Comparison of GEPs revealed that 266 genes were upregulated, and 345 genes were downregulated. Part of a heat map (the top 30 genes downregulated and upregulated by EHMT2) is shown in **Figure 6**. CD5 and CCND1 were both found to be downregulated in response to *EHMT2* knockdown. KEGG analysis revealed that Tec kinase, IL-8, B-cell antigen receptor (BCR), IL-2, and Ephrin B signaling pathways were significantly inhibited after *EHMT2* knockdown. The top 5 enriched signaling pathways and regulated genes are shown in **Table 1**.

**Table 1:** The top 5 enriched signaling pathways and regulated genes after the knockdown of *EHMT2* in mantle cell lymphoma cases

Pathways	Genes
Tec kinase signaling	VAV2, RHOC, GNB5, MAPK9, NFKB1, PAK1, LCK, GNG11, VAV3, LYN, HCK, MAPK10, RHOF, JAK3, FNBP1, ITGA4, PRKCA, PRKCB
IL-8 signaling	RAC2, FLT1, RHOC, GNB5, MAPK9, IRAK3, NFKB1, CCND1, VEGFA, ROCK2, GNG11, MAPK10, RHOF, NFKBIB, FNBP1, PRKCB, PRKCA
B Cell receptor signaling	VAV2, RAC2, PTPN6, POU2F2, MAPK9, NFKB1, SYNJ2, CFL2, CDC42, VAV3, LYN, NFATC2, MEF2C, NFKBIB, MAP3K2, PRKCB
IL-2 signaling	VAV2, TGFBR1, TGFB1, VAV3, MAPK10, MAPK9, SMAD4, NFATC2, NFKBIB, NFKB1
Ephrin B signaling	VAV2, ROCK2, RAC2, PAK1, GNG11, CFL2, CDC42, VAV3, GNB5

Next, we compared GEP data with the DNA methylation profiles of EHMT2 + MCLs. Notably, some differentially expressed genes after* EHMT2* knockdown were aberrantly methylated in EHMT2 + MCL cases. Among those genes, *MARCH1*, *CCDC50*, *STXBP6*, *SH3KBP1*, *HIP1*, *SLC39A11*, *NEDD4L*, *WNT3,* and *ACPP* genes were hypomethylated and were downregulated in response to *EHMT2* knockdown. However, the *TRPC6* gene was hypermethylation and upregulated.

## Discussion

H3K9 methylation and DNA methylation are tightly associated with heterochromatin formation and transcriptional repression. In *Neurospora*, H3K9 methylation controls DNA methylation^22^. In *Arabidopsis*, DNA methylation and H3K9 methylation are functionally interdependent^23^. There are also functional links between H3K9 methylation and DNA methylation in mammals^24,25^. SUV39H1/2 catalyzes the trimethylation of H3K9 and is necessary to maintain DNA methylation^26,27^. Dimethylation of H3K9 plays an equally important role in gene silencing and is catalyzed by distinct H3K9 methyltransferases, EHMT2 and EHMT2-like protein (EHMT1/GLP)^28,29^. EHMT2 is overexpressed in many types of cancers, and high EHMT2 expression is associated with a poor prognosis^13,15^. EHMT2 has been demonstrated as a central control point of the immune system in T-cell development, differentiation, and function, particularly in naïve T cells, and H3K9me2 and EHMT2 act as additional layers of negative regulation to maintain cells in a naïve state^30^. However, in contrast to T cells, previous studies have suggested that EHMT2 may be dispensable for normal B cell development and has little effect on most B cell functions^31,32^. In our study, EHMT2 was not present in any RH cases but was activated in 68.57% of MCL cases, indicating that EHMT2 may play a crucial role in the malignant transformation of mantle cells.

Recent reports using genome-wide approaches are changing our understanding of the role of DNA methylation in cancer^33,34^. In the current study, we identified a substantial number of differentially methylated genes in EHMT2 + MCL cases, including both previously reported and novel methylated genes, with a wide range of functions. *HOX* genes (*n* = 15) were common targets of methylation in our study, consistent with reports by Kanduri et al., who identified 13 differentially methylated HOX genes in MCL^35^. In addition to *HOX* genes, *FOX*, *SOX*, and *PAX* genes were found to be frequently methylated in EHMT2 + MCL cases. Among these genes, *SOX11* and *PAX5* were hypomethylated, consistent with their protein expressions in MCL. Our data provided a possible mechanism for SOX11 and PAX5 activation in MCL. Furthermore, we provided the potential roles of other members of these families in MCL.

Previous reports have suggested that genetic and epigenetic alterations in cancer may affect the same genes and pathways^36,37^. In our results, some of the differentially methylated genes in EHMT2 + MCL cases have also been reported with recurrent genetic alterations in lymphoma, such as *ALK*, *DUSP22*, *NCOR2*, *RORA*, *DLC1*, *JAG1/2*, *JAK1*, *NOTCH4,* and *CD19*^38–45^. Our study showed that DNA methylation changes in these genes occurred less frequently at promoters and CGIs, but were instead largely targeted within gene-body regions.

We also investigated the function of EHMT2 in MCL cases. Inhibition of EHMT2 enhanced apoptosis and induced G0/G1 arrest in MCL cells. Fukuda et al. reported that EHMT2-dependent histone methylation was induced in the G1 phase of the cell cycle^46^. More recently, cyclin D1 was reported to be required for the recruitment of EHMT2 to target genes in chromatin and for H3K9 dimethylation^47^. In our study, the EHMT2 inhibitor, BIX01294, downregulated the expression of cyclin D1 by inhibiting the levels of H3K9me2 and H3K27me2, and GEP data showed the downregulation of CCND1 after *EHMT2* knockdown, supporting the cooperation between EHMT2 and cyclin D1. In addition to cyclin D1, cell cycle proteins, CDK4 and p21, were also downregulated after EHMT2 inhibition, and accordingly, apoptosis-related proteins Bax and capase-3 were upregulated. Notably, besides CCND1, CD5 was also downregulated after *EHMT2* knockdown, indicating the important regulatory role of EHMT2 in the development of MCL. Bruton tyrosine kinase (BTK), a member of the Tec tyrosine kinases family, plays a central role in B cell lymphomas, and BTK inhibitors are increasingly replacing chemotherapy-based regimens, particularly in patients with chronic lymphocytic leukemia and MCL. In our study, we found that EHMT2-regulated genes were most enriched in Tec kinase (another member of the Tec tyrosine kinases family) signaling pathway. Furthermore, by comparing GEP data with the methylation profiles in EHMT2 + MCL cases, we identified several methylated genes regulated by EHMT2. It should be mentioned that after treatment with BIX01294, not only the dimethylation of H3K9 was inhibited, but also the dimethylation of H3K4 was increased, confirming previous reports that H3K4me2 and K3K9me2 showed opposite effects in regulating some genes transcription^48,49^.

In epigenetic regulatory networks, various modification enzymes often interact with each other or form complexes to regulate gene expression. The E3 ubiquitin ligase UHRF1 is a master regulator of epigenetic modifications due to its ability to recognize modifications of both DNA and histones. By recognizing hemi-methylated DNA, UHRF1 maintains genomic DNA methylation by recruiting DNA (cytosine-5)-methyltransferase 1 (DNMT1) to DNA replication sites. In our study, we demonstrated that UHRF1 worked together with EHMT2 in MCL cells, consistent with previous results in other tumors. Direct cooperation between EHMT2 and DNMT1 during cell division has been reported^50^. However, whether EHMT2 exhibits cross-talk with HDACs is unclear. Our results revealed that EHMT2 interacted with HDAC1 and HDAC2, but not with HDAC3 in MCL, providing a mechanism of coordinated EHMT2 and histone acetylation during the development of MCL.

## Conclusions

In summary, EHMT2 was overexpressed in MCL and involved aberrant methylation of multiple members including the HOX, FOX, PAX, SOX, and CDX families. EHMT2 could work together with HDAC1/HDAC2 and UHRF1 to regulate the expression of multiple genes, which were mostly enriched in the Tec kinase signaling pathway. Our observations highlighted EHMT2 as a potential novel target for epigenetic-based therapy in MCL patients.
