# Supplementary material for: EHMT2 (G9a) activation in mantle cell lymphoma and its associated DNA methylation and gene expression
Source: Cancer Biol Med. 2021 Oct 12;19(6):836–49. doi: 10.20892/j.issn.2095-3941.2020.0371 (PMC9257318; doi:10.20892/j.issn.2095-3941.2020.0371)
Supplement: Supplementary file 1 [file cbm-19-836-s001.pdf]

Supplementary materials

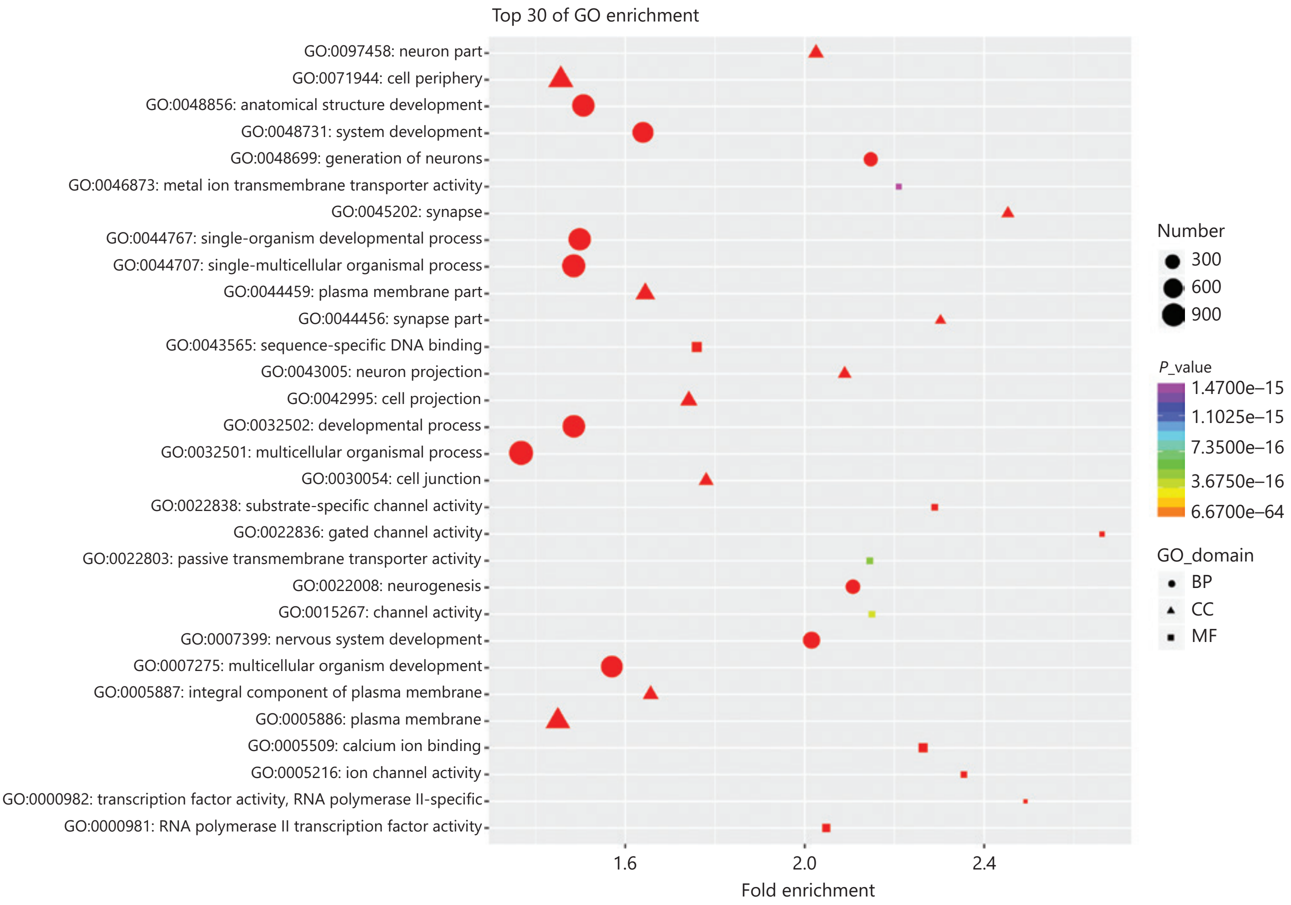

Figure S1 Gene Ontology (GO) analysis of differentially methylated genes in EHMT2 + mantle cell lymphoma cases (β > 0.14; *P* < 0.05). The x-axis, enrichment factor; y-axis, the GO category. Red denotes small *P*-values and green denotes larger values. A round node denotes a biological process; the triangle denotes a cellular component and a square denotes a molecular function. The top 30 GO terms are shown.

Table S1 The correlation between EHMT2 expression and the clinicopathological features in EHMT2 + mantle cell lymphoma cases

Clinicopathological feature	EHMT2 (+)	EHMT2 (-)	*P*
	n	*n*	
Age (year)			0.281
≤60	12	3	
>60	12	8	
Sex			0.387
Male	17	10	
Female	7	1	
Ki67			0.265
≤30%	17	5	
>30%	7	6	
LDH*			0.126
≤430	0	2	
>430	19	9	
Ann Arber stage			0.684
1	8	2	
II	6	4	
III	1	1	
IV	9	4	

*Five patients did not have detectable LDH levels.

Table S2 The frequency distribution of all significantly differentially methylated CpG sites in EHMT2 + mantle cell lymphoma cases

Methylation (β) difference	Hypermethylated CpG Sites *n* (%)	Hypomethylated CpG Sites *n* (%)	Total CpG Sites *n* (%)
≥40%	685 (51.20)	2,859 (32.51)	3,544 (34.97)
≥30–<40%	653 (48.80)	5,936 (67.49)	6,589 (65.03)
Total	1,338	8,795	10,133

Table S3 The distribution of genomic regions for all significantly differentially methylated CpG sites in EHMT2 + mantle cell lymphoma cases compared with those in reactive hyperplasia cases

Genomic region of CpG sites	All CpG sites	Hypermethylation	Hypomethylation
		CpG Sites *n* (%)	CpG Sites *n* (%)
CpG Island (CGI)	161,598 (18.64)	1,112 (83.11)	98 (1.11)
Shore (0–2 kb from CGI)	154,735 (17.85)	161 (12.03)	766 (8.71)
Shelf (4–6 kb from CGI)	61,811 (7.13)	15 (1.12)	542 (6.16)
Open sea	488,751 (56.38)	50 (3.74)	7,389 (84.02)
Total	866,895	1,338	8,795

Table S4 The biological characteristics of significant CpG sites identified in EHMT2 + mantle cell lymphoma cases

Features	Significantly hypermethylated CpG sites	Significantly hypomethylated CpG sites
Infinium		
I	869	439
II	469	8,356
Functional genome distribution		
Promoter	486	758
Body	270	3,601
3'UTR	16	150
Intergenic	296	3,742
Phantom promoter		
High CpG content	193	1
Low CpG content	5	24

Table S5 The top 30 significantly hypomethylated genes in EHMT2 + mantle cell lymphoma cases compared with those in reactive hyperplasia cases

CpG site	Mean MCL tissue	Mean RH tissue	Mean difference	Diffscore	Hypomethylated gene
cg06377160	0.8462059	0.2192933	−0.6269127	−75.48992	SNX19
cg02592727	0.4547865	0.1461613	−0.3086252	−57.0156	RDH13
cg25535027	0.487113	0.2483802	−0.2387328	−52.81252	SLC39A7
cg04940054	0.507884	0.2446689	−0.2632152	−52.20582	LINC00939
cg10456990	0.4365879	0.1763136	−0.2602743	−51.72263	SIM2
cg11369071	0.7074636	0.3901251	−0.3173386	−51.3016	LOC100270746; C6orf41
cg23412091	0.6139717	0.2862514	−0.3277203	−50.02227	TMEM41A
cg11733675	0.5292104	0.2935199	−0.2356906	−49.86818	NLGN1
cg22717227	0.4211193	0.1759834	−0.2451359	−49.55123	C10orf53
cg10938233	0.5663867	0.3462509	−0.2201358	−49.11399	CSMD1
cg24487279	0.4838578	0.07269558	−0.4111622	−48.73317	OXR1
cg22204479	0.590589	0.1327952	−0.4577938	−48.49442	NKX2-4
cg22982368	0.4318252	0.08350327	−0.3483219	−48.24937	PAX6
cg11708616	0.4562688	0.08895898	−0.3673099	−47.4348	FOXQ1
cg10648197	0.4631557	0.1696359	−0.2935198	−47.23201	SPAG6
cg16937769	0.4428068	0.204212	−0.2385948	−46.59911	HOXC4; HOXC6; HOXC5
cg23622153	0.4364784	0.1025535	−0.3339249	−45.83541	DSEL; LOC643542
cg05181186	0.7016985	0.05343278	−0.6482657	−45.39481	CDS1
cg05563287	0.5781229	0.2386259	−0.339497	−45.30098	ADAMTS17
cg19024344	0.6073342	0.1628704	−0.4444638	−45.18105	DLC1
cg18198999	0.4914254	0.1786578	−0.3127676	−45.05529	DMRTA2
cg18063017	0.5308674	0.2182848	−0.3125826	−45.03379	LHX4
cg24925400	0.5650398	0.288571	−0.2764688	−44.78916	PITX2
cg07588113	0.354783	0.09480181	−0.2599812	−44.6541	C7orf63
cg23649435	0.5065351	0.06638894	−0.4401462	−43.82687	STOX2
cg04524652	0.4272011	0.05893176	−0.3682693	−43.45611	FAM160A1
cg20250341	0.4396492	0.2382616	−0.2013876	−43.34166	C17orf44
cg22299454	0.4504218	0.116345	−0.3340768	−43.26916	MARCH1
cg19909787	0.4934257	0.1125836	−0.3808421	−43.01773	HMX2
cg04390523	0.5996584	0.1323086	−0.4673498	−42.95142	EDNRB

Table S6 The top 30 significantly hypermethylated genes in EHMT2 + mantle cell lymphoma cases compared with those in reactive hyperplasia cases

CpG site	Mean MCL tissue	Mean RH tissue	Mean difference	Diffscore	Hypermethylated gene
cg23230830	0.3178573	0.7388198	0.4209625	60.75093	LOC646627
cg11236641	0.1689083	0.7677054	0.5987971	56.51852	PRDM16
cg25224813	0.1847852	0.604936	0.4201508	56.05684	PDGFC
cg10499172	0.1291039	0.4468592	0.3177553	55.1452	DCPS
cg12770539	0.1585084	0.5785133	0.4200049	53.87254	EEPD1
cg25835179	0.1381735	0.444884	0.3067105	53.15146	ACY3
cg09184271	0.1979736	0.4432445	0.2452709	52.77735	LOC102724188
cg10572188	0.4168094	0.7306411	0.3138317	52.0444	CCDC52
cg06581818	0.4698206	0.6787459	0.2089253	51.51563	CEACAM20
cg26935044	0.1929822	0.6279361	0.4349538	51.24723	CCL4
cg00869927	0.1722254	0.5451878	0.3729625	50.25766	SGIP1
cg09740425	0.4776053	0.7732241	0.2956189	49.49221	MED12L
cg20819895	0.1519993	0.4424257	0.2904264	48.20529	MYO1D
cg02643433	0.1653855	0.4360989	0.2707134	48.18273	DSP
cg09163930	0.1953747	0.533519	0.3381443	47.71744	PDGFRB
cg14514130	0.4666558	0.7356675	0.2690116	47.49101	NFASC
cg02935847	0.2580396	0.6690312	0.4109915	47.19162	CELF6
cg19555191	0.376651	0.6056705	0.2290194	46.91182	DPP6
cg07395331	0.5114742	0.7993112	0.2878371	46.90426	PPM1H
cg12166899	0.3803716	0.7885495	0.4081779	46.54666	JAG2
cg07082484	0.2330821	0.5330534	0.2999713	46.53505	SH3RF1
cg01258789	0.2288738	0.5497532	0.3208793	46.28978	CRB1
cg19007141	0.4268272	0.7196074	0.2927801	46.27856	MAML3
cg03610280	0.1651199	0.4927088	0.3275889	46.23247	ENTPD1
cg21718826	0.4849877	0.7668172	0.2818294	46.19204	NUDT16L1
cg00632811	0.09330513	0.5071465	0.4138414	46.17984	PWP1
cg07579107	0.1863194	0.5948922	0.4085729	46.07104	WDR72
cg14235273	0.5881255	0.8547997	0.2666742	45.98321	ABI3
cg14454798	0.223995	0.4932028	0.2692078	45.86175	CPEB4
